# Fabrication of hybrid graphene oxide/polyelectrolyte capsules by means of layer-by-layer assembly on erythrocyte cell templates

**DOI:** 10.3762/bjnano.6.237

**Published:** 2015-12-04

**Authors:** Joseba Irigoyen, Nikolaos Politakos, Eleftheria Diamanti, Elena Rojas, Marco Marradi, Raquel Ledezma, Layza Arizmendi, J Alberto Rodríguez, Ronald F Ziolo, Sergio E Moya

**Affiliations:** 1 Soft Matter Nanotechnology, CIC biomaGUNE, Paseo Miramón 182 C, 20009 San Sebastian, Spain; 2 Departamento de Materiales Avanzados, Centro de Investigación en Química Aplicada, Blvd. Enrique Reyna Hermosillo No.140 C.P. 25294 Saltillo, Coahuila, México

**Keywords:** capsules, erythrocytes, graphene, layer by layer, polyelectrolyte membranes

## Abstract

A novel and facile method was developed to produce hybrid graphene oxide (GO)–polyelectrolyte (PE) capsules using erythrocyte cells as templates. The capsules are easily produced through the layer-by-layer technique using alternating polyelectrolyte layers and GO sheets. The amount of GO and therefore its coverage in the resulting capsules can be tuned by adjusting the concentration of the GO dispersion during the assembly. The capsules retain the approximate shape and size of the erythrocyte template after the latter is totally removed by oxidation with NaOCl in water. The PE/GO capsules maintain their integrity and can be placed or located on other surfaces such as in a device. When the capsules are dried in air, they collapse to form a film that is approximately twice the thickness of the capsule membrane. AFM images in the present study suggest a film thickness of approx. 30 nm for the capsules in the collapsed state implying a thickness of approx. 15 nm for the layers in the collapsed capsule membrane. The polyelectrolytes used in the present study were polyallylamine hydrochloride (PAH) and polystyrenesulfonate sodium salt (PSS). Capsules where characterized by transmission electron microscopy (TEM), atomic force microscopy (AFM), dynamic light scattering (DLS) and Raman microscopy, the constituent layers by zeta potential and GO by TEM, XRD, and Raman and FTIR spectroscopies.

## Introduction

The past decades have witnessed explosive growth in research on low-dimensional carbon forms with graphene and carbon nanotubes in the forefront [1–3]. The unique electrical, mechanical, thermal and optical properties of graphene in particular, and of its derivatives, continue to be explored theoretically and experimentally in physics, chemistry, engineering and biology with emerging potential applications of societal importance [4–7]. The unique properties of graphene (G), which forms super strong sheets of carbon a single atom thick [8], result from its planar nature and the sp^2^ hybridization of its carbon atoms.

Single layer, bi- and few-layer graphene are difficult to work with in soft matter or wet chemical applications because of dispersibility issues and the tendency to form multi-layered agglomerates, which begin to acquire the properties of graphite [9–11]. Because of these difficulties, most studies of graphene, whether for layered assembly or other investigations, have been performed on graphite oxide or its exfoliated form, graphene oxide (GO), which bears a mix of sp^2^ and sp^3^ hybridized carbons in an overall planar structure. These derivatives of graphene can also possess unique and often controllable properties and have the potential to be reduced to what is called a reduced form of graphene oxide, rGO, by chemical or physical means, which can lead to materials with properties more like G than GO [12–15]. The derivatization of G to form GO leads to easily dispersible and stable systems containing GO with an overall surface charge while exfoliated, for example, in water [16]. The use of GO sheets in the formation of hierarchical structures and assemblies is a subject of current interest, and if done by procedures involving wet chemical techniques, offers much potential for the development of advanced and composite layered materials.

The assembly of GO in thin films on the basis of its surface charge can be accomplished using the layer-by-layer technique (LbL). Although the technique was originally developed for the alternating assembly of polyelectrolytes (PE) of opposite charge, LbL can be and has been extended to include particles, 2D layered materials, nanostructures, and lipid vesicles, which provide charged surfaces, or, which can have complementary interactions with another material that can lead to a subsequent or alternating assembly [17]. There are examples in literature of the assembly of GO with polycations using LbL as a promising tool for the incorporation of GO into nanoscale organized thin films and the subsequent electrochemical reduction of the GO to rGO [18–19].

Using LbL, we explored the assembly of exfoliated GO into 3D structures and developed micrometre-sized capsules on the basis of GO and polyelectrolytes using chicken erythrocyte cells as templates. Template cells have been used in the past as capsule templates on the basis of a simple protocol, which includes the oxidation of the cell components to leave a thin polymer film in the form of a capsule that mimics the dimensions and topological features of the cell template [20–22]. We show that by incorporating GO within the LbL assembly on top of the erythrocytes, 3D closed films containing walls of PE/GO and an empty volume can be produced.

Capsules based on GO can have applications in drug delivery, especially for topical applications, as the LbL capsules provide low degradability and high stability through the GO. The use of GO in the fabrication of capsules was reported by J. Hong et al. [23] and by Kurapati et al. [24], the last one with a focus on drug delivery. The authors, however, use a different protocol and templates for their fabrication. In the present work, we produce continuous GO capsules with the shape of cells and show the presence and location of the GO by Raman confocal microscopy. Another potential application of these capsules is for the production of PE/GO films with a high concentration of GO, which was achievable in the present study by employing a high concentration of exfoliated GO during the layer by layer assembly. This is due to the coating protocol employed in which the cells are suspended in a GO solution and then centrifuged. Under these conditions, a high concentration of GO is achieved on the cell surface leading to a continuous coating.

Once dried, the capsules collapse by losing internal volume and form planar films with nanoscale thicknesses. Such films could be used to build additional hierarchical structures or for integration into devices; moreover, by the judicious choice of reagents, the GO may be further reduced to form rGO to alter particularly the electronic, mechanical and optical properties of the films.

## Results and Discussion

**Figure 1:**
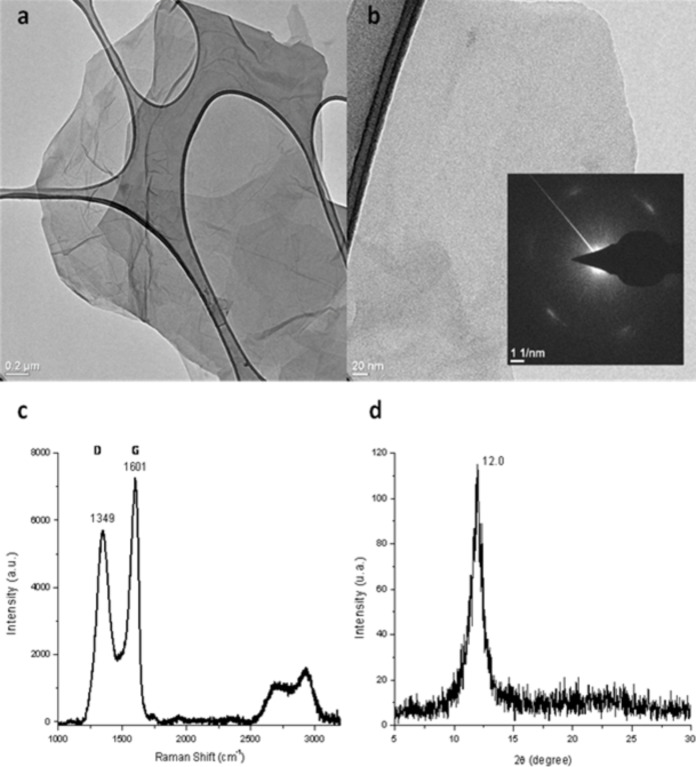
GO data; a) and b) TEM images of graphene oxide on lacey carbon, inset: SAED showing diffraction spots of hexagonal patterns; c) Raman spectrum with 532 nm excitation d) XRD pattern showing (002) with *d* = 0.737 nm.

Graphene oxide was prepared by the strong acid oxidation of powdered graphite by a modified Hummer’s method [25–26]; characterization data are shown in Figure 1. Multiple TEM images formed from exfoliated GO in water and dried on lacey carbon grids show micrometre-size, few-layer GO sheets with some gentle folds and ripples (Figure 1a,b). The SAED inset in panel b shows diffraction spots indicative of hexagonal patterns [27]. Raman spectroscopy (Figure 1c) shows the expected prominent peaks for GO at 1349 and 1601 cm^−1^, with a D/G ratio of 0.8. The D resonance corresponds to the vibration of sp^2^ carbon while G corresponds to sp^3^ carbon and defects associated with vacancies and grain boundaries [28]. The peaks at higher wavenumbers are also characteristic of graphene oxide. XRD results (Figure 1d) indicate the absence of graphite and show the prominent (002) peak at 12.0 degrees 2θ, indicating an inter-planar GO sheet distance of 0.737 nm [29]. Prominent FTIR bands for GO were observed at 3415, 1723, 1623, 1394 and 1050 cm^−1^ as reported in [30].

**Figure 2:**
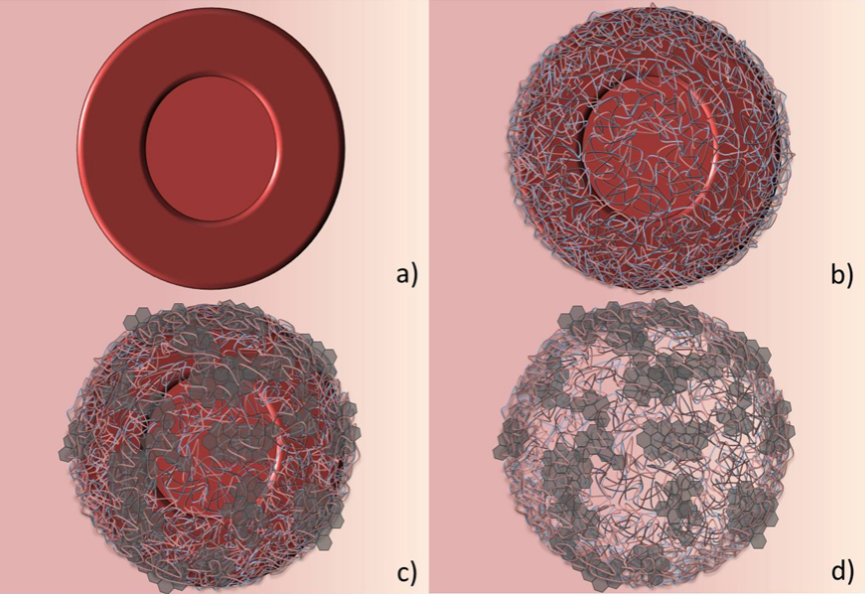
Schematic illustrations of a) the glutaraldehyde fixed red blood cells, b) the fixed erythrocytes coated with four layers of PSS/PAH, c) the fixed cells in (b) coated with additional GO/polyelectrolyte layers and d) the hybrid GO/polyelectrolyte capsule after NaOCl oxidation of the cell.

A scheme of the layer-by-layer assembly applied on the erythrocytes for the deposition of GO and polymers, as well as for the subsequent NaOCl oxidation and capsule generation, is represented in Figure 2. In the first step, the red blood cells are crosslinked with glutaraldehyde. The crosslinking is necessary to insure that the polyelectrolytes will not disrupt the erythrocyte membrane. Erythrocytes were chosen because of their simple structure that lacks a nucleus, which makes the degradation of the whole cell content with NaOCl solution easier. Before the coating with GO, four layers of PSS/PAH were assembled on top of the cell. Despite the erythrocytes have a slightly negative charged surface the first assembled layer was PSS, which is also negative. We have observed earlier that the PSS assembles better as first layer on the cells causing significantly less aggregation of the cells [19]. The coating of the cells with an initial film of PSS/PAH is done to ensure a homogenously charged surface prior to the GO deposition. The assembly of the polyelectrolytes in the first layers and between the GO was done in 0.5 M NaCl as in these salt conditions the polymers assemble in a denser and compact structure, due to the optimal coiled conformation of the PEs at this ionic strength.

The assembly of exfoliated GO was done in water at pH 10 to avoid the screening of the charges on the GO. If present, screening the GO charges could lead to a stronger interaction between GO sheets in solution leading to aggregation. Charge screening would also eventually reduce the electrostatic interaction between the GO and the polyelectrolyte on the cell, which would be unfavourable for assembly. Between each GO deposition, we deposited either one layer of PAH or three layers of PAH/PSS/PAH. Since the assembly of one layer of PAH between the GO layers may not be sufficient to lead to a complete coating of the surface and full recharging, we additionally prepared capsules assembling three polyelectrolyte layers in between the GO layers. The addition of three polyelectrolyte layers on top of the GO layer makes the procedure more time consuming but we observe a major improvement in the stability of the coated cell, thus reducing aggregation.

The removal of the cell interior by NaOCl is based on the oxidation of the cell content, which is mainly proteins, which become defragmented and are easily eliminated during centrifugation and washing cycles with NaCl and water. Oxidation also affects the PAH within the layers but a thin polymer film remains as was shown previously [31]. The possible oxidation of the GO with NaOCl would increase the percentage of negative charge on the GO surface.

**Figure 3:**
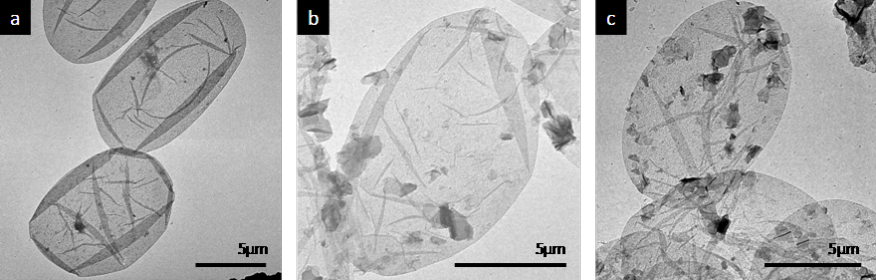
TEM micrographs of a) sample 1: (PSS/PAH)_4.5_^0.5M^, b) sample 2: (PSS/PAH)_2_^0.5M^ + [GO/(PAH/PSS/PAH)^0.5M^ /GO] + (PSS/PAH)^0.5M^, c) sample 3 (PSS/PAH)_2_^0.5M^ + [GO/PAH]_2.5_ + (PSS/PAH)^0.5M^ using a concentration of 0.1 mg/mL of GO during the assembly.

The morphology and structural characteristics of the capsules were first studied by TEM which also provided proof of the core removal. Figure 3a shows an image of the capsules prepared as a control which do not contain GO. They clearly retain the shape of the erythrocytes used as templates. The capsules contain only water and collapse in the dry state to ellipsoid shaped films. The drying process is responsible for the appearance of edges on the capsule surface.

In Figure 3b,c we observe two examples of capsules fabricated with GO at a 0.1 mg/mL concentration with one PAH layer and three PAH/PSS/PAH layers in between the GO. As for the polyelectrolyte capsules, the GO capsules also retain the shape and size of the cells used as templates. The GO capsules are also empty as they are in a collapsed state after drying. On the capsule wall we can recognize the presence of a structure of patches, with dimensions between a few hundred of nanometers and one micrometre. GO is recognized in the structure but embedded within the polyelectrolyte materials. GO is present on some random spots in the capsule, the rest of the capsule structure is very similar to the control capsules. The patches have the characteristic of GO sheets in the range of micrometres.

**Figure 4:**
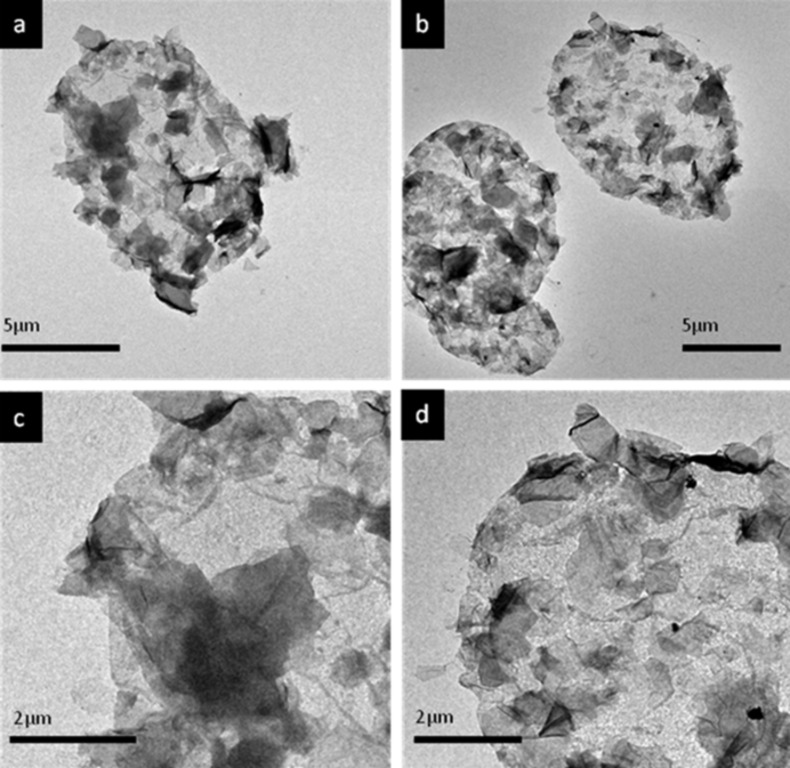
TEM micrographs of a) sample 2: (PSS/PAH)_2_^0.5M^ + [GO/(PAH/PSS/PAH)^0.5M^/GO] + (PSS/PAH)^0.5M^, b) sample 3: (PSS/PAH)_2_^0.5M^ + [GO/PAH]_2.5_ + (PSS/PAH)^0.5M^, c and d) magnification of samples 2 and 3, respectively. GO concentration was 0.2 mg/mL.

We decided to increase the concentration of GO to 0.2 mg/mL in order to increase the GO coating. For this GO concentration TEM imaging reveals that indeed the GO coating has become visible all over the surface of the capsules as can be appreciated in Figure 4.

In Figure 4a,b the capsules are prepared as in Figure 3a,b but with a GO concentration of 0.2 mg/mL instead of 1 mg/mL. Figure 4c,d are magnifications of a selected area from the corresponding capsules represented in Figure 4a,b, respectively. The GO sheets can be recognized all over the surface. Presumably, there is more GO deposited in the areas that show more contrast. The presence of these darker areas could imply that there may be GO stacking on top of previously assembled GO but still separated by PEs. The TEM images also confirm that by increasing the GO concentration, there is a complete coverage of the capsules with GO, which is not observed at the concentration of 1 mg/mL. The TEM images also proves the formation of a complete and continuous coating of the capsule surface with GO with only three layers of GO being assembled, which, interestingly, is not obtained for the same number of layers on a planar surface. The use of a layer of PAH or three polyelectrolyte layers between the GO assemblies does not seem to affect the integrity of the capsules. Accordingly, we proceeded with the assembly with just one intermediate polyelectrolyte layer, which results in a reduced number of steps during the assembly for ease of fabrication.

**Figure 5:**
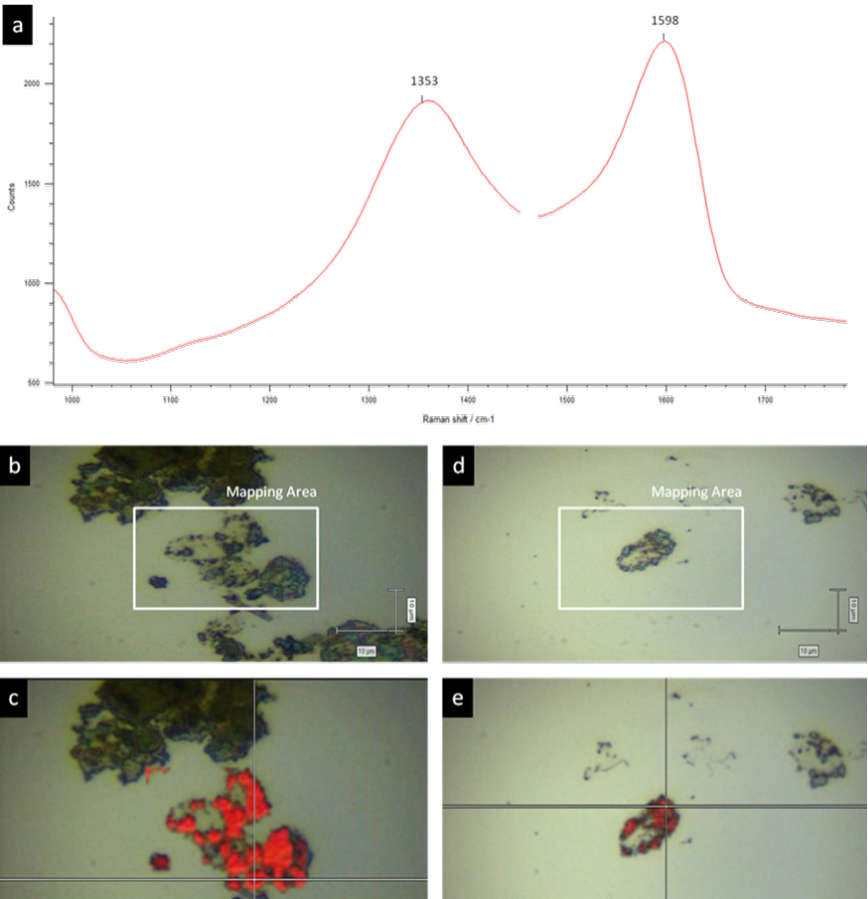
Raman of the GO-capsules. a) Raman spectra of GO sheets, G band located at 1598 cm^-1^ and D band at 1353 cm^-1^. b) Image of the sample 2: (PSS/PAH)_2_^0.5M^ + [GO/(PAH/PSS/PAH)^0.5M^ /GO] + (PSS/PAH)^0.5M^ capsules onto Si wafer with the selected area to be analysed. c) Mapping by intensity of the G band at 1598 cm^-1^ of the area selected in b. d) Image of sample 3 (PSS/PAH)_2_^0.5M^ + [GO/PAH]_2.5_ + (PSS/PAH)^0.5M^ capsules onto Si wafer with the selected area to be analysed. e) Mapping by intensity of the G band at 1598 cm^-1^ of the area selected in d.

Although the GO sheets can be recognized by their characteristic shape, we performed confocal Raman microscopy imaging to provide additional proof that the patches on the capsule wall correspond to GO. A representative scan of one capsule is shown in Figure 5a.

The surface of the capsule was scanned point by point. At each point, the Raman spectra revealed the presence of characteristic and strong bands at 1353 and 1598 cm^−1^, which are characteristic for the GO and graphene structures and which cannot be detected in the capsules prepared solely with polyelectrolytes. The peaks in the Raman spectra of the capsules correspond to the G and D bands observed in the pristine GO (vide supra), with the typical shift in the G band to higher frequencies for GO with respect to graphite [28].

**Figure 6:**
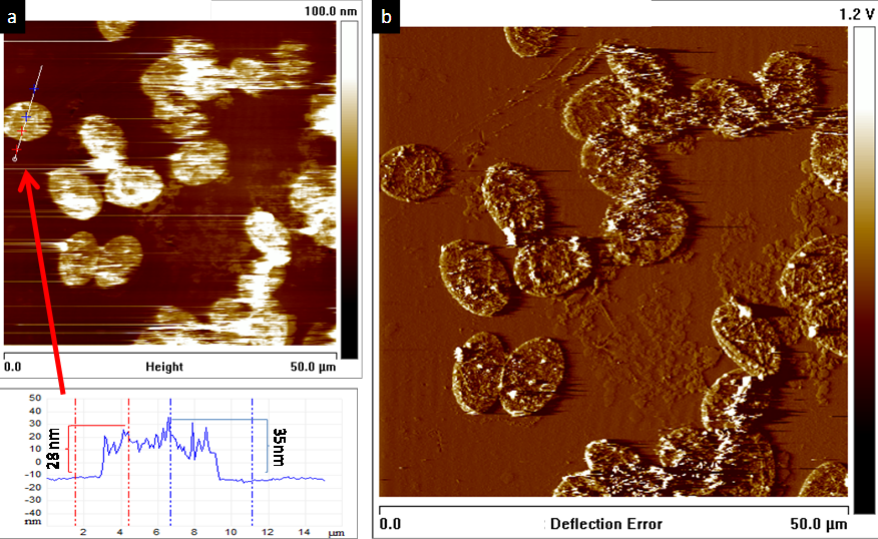
Atomic force microscope images of dried hybrid PE/GO capsules. a) Height image of a 50 × 50 µm scan, with a profile corresponding to the line drawn in the image. b) Deflection image of the same area analysed in a).

In order to determine the thickness of the capsule walls we performed AFM imaging of the capsules in the collapsed state. From the AFM images (Figure 6a) an estimated maximal thickness of about 30 nm could be determined for the capsules, implying a thickness of the order of 10–19 nm for the walls of the capsule in the dry state. These values are obtained assuming that the thickness of the collapsed capsule is twice the thickness of the capsule wall since the capsule loses internal volume when drying. These values are taken from the maximal and minimal thickness measured for the capsule. AFM imaging also confirmed the stability of the capsules, which retain their shape and integrity after collapsing in air (Figure 6b).

## Experimental

### Materials

Polyallylamine hydrochloride, (PAH, *M*_w_ 15 kg/mol), polystyrenesulfonate sodium salt, (PSS, *M*_w_ 70 kg/mol), sodium hypochlorite with active chlorine 13%, phosphate buffered saline 10× (PBS), glutaraldehyde solution grade II 25% in water, Hank´s balanced salt solution 10×, sodium chloride and graphite powder (<45 μm, ≥99.99% trace metals basis) were obtained from Sigma–Aldrich. Lymphocytes isolation solution (Ficoll) was purchased from Rafer Zaragoza, Spain. Full blood chicken collected in Alsever's anticoagulant solution was purchased from Harlan Laboratories Models S.L., Barcelona, Spain.

### Methods

#### Erythrocytes

Erythrocytes were separated from the chicken blood and the rest of the plasma components by centrifugation. Chicken blood conserved in Alsever´s solution was diluted with (Ca, Mg) Hank´s solution and carefully poured onto a Ficoll solution to avoid mixing. Afterwards the resulting solution was centrifuged at 800*g* for 30 min at 4 °C depositing the erythrocytes at the bottom and the rest of the plasma in different phases above them. Only the erythrocytes were obtained and cleaned twice with Hank´s solution by centrifugation under the same conditions as before. Cells were then fixed for 1 h at 4 °C with glutaraldehyde at a concentration of 2.5% in filtered PBS buffer. After fixation, they were centrifuged and washed in 0.9% NaCl several times at 10 °C and kept refrigerated.

#### Graphene oxide

Graphene oxide was prepared by strong acid oxidation of graphite using Hummer’s method [25] as modified in [26] where the dispersion was water washed to neutral pH. Following this, we washed with dilute HCl followed again by water washes to neutral pH. The graphene oxide was exfoliated via ultra-sonication and microwave irradiation. FTIR spectra were taken with a Nexus 470 Spectrometer over 4000–400 cm^−1^ using multiple scans and 4 cm^−1^ resolution in transmission mode.

#### Layer-by-layer assembly

The LbL assembly of GO and polyelectrolytes was performed on the fixed erythrocytes. Previous to the GO assembly, two bilayers of PSS/PAH were assembled. The polyelectrolytes were assembled at a concentration of 1 mg/mL in 0.5 M NaCl. Two washings were performed with water between each layer assembly by centrifugation at 67.08*g* for 2 min. The first assembled layer on top of the cells was always PSS since it was already shown that it assemblies better using a polyanion rather than a polycation on the erythrocytes, despite the negative charge of the cells [32]. After the fourth layer was assembled, GO is deposited in a concentration of 0.1 or 0.2 mg/mL in water (pH 10). The assembly of GO is performed without NaCl as the salt would screen the charges of the GO and lead to aggregation. The incubation time for polyelectrolytes and GO was always 2 min. After GO deposition, the cells were also washed in water by centrifugation. GO was assembled alternating with PAH or with three polyelectrolyte layers; PAH/PSS/PAH, until a total number of eleven layers was achieved in both cases. Also, another sample with just nine layers of PEs, without GO, was assembled as a control, following the same procedure described above.

#### Capsule fabrication

Once the GO/polyelectrolyte multilayer was assembled on top of the cells, they were exposed to aqueous 1% NaOCl. The oxidizing solution degrades the cells by breaking their protein constituents through oxidation. After NaOCl treatment, samples were washed first in NaCl and then in water for several cycles to remove the products of oxidation.

#### Electron transmission (TEM) and X-ray diffraction measurements

Transmission electron microscopy images were taken using a JEOL JEM-1400PLUS (40–120 kV) equipped with a GATAN US1000 CCD camera. An aliquot of 5 µL of each sample was deposited on grids of carbon film 400 mesh Cu, (50/bx) and left to evaporate. The grid was previously stained with uranyl acetate.

TEM imaging of the GO was done using an FEI Titan 80–300 kV field emission gun microscope, which has a symmetrical condenser-objective lens type S-TWIN (with an spherical aberration *C*_s_ ≈ 1.25 mm). The images were acquired with a CCD camera of samples prepared from water dispersions cast on lacey carbon grids.

#### X-ray diffraction

XRD data for GO were obtained using a Siemens D-5000 operated at 35 kV and 25 mA and Cu Kα radiation.

#### Raman spectroscopy and confocal Raman microscopy

Raman analysis was performed using a Renishaw inVia Raman microscope with a 100× objective, a 532 nm laser excitation and a 1800 mm^−1^ grating. The system was calibrated to the spectral line of crystalline silicon at 520.7 cm^−1^. A drop of a diluted solution containing the capsules was placed onto a silicon wafer and left evaporating overnight.

#### Atomic force microscopy (AFM)

Atomic force microscopy studies were performed using a Veeco Multimode AFM attached to a Nanoscope V controller. The samples were imaged in contact mode in air, using a TESP-V2 silicon probe with *k* = 42 N/m. A drop of a diluted solution with the capsules was placed on a glass substrate with a thin layer of Au deposited on top. The sample was left evaporating and afterwards imaged.

## Conclusion

We have shown that 3D micrometre-sized objects in the form of capsules can be fabricated on the basis of the LbL assembly of GO and polyelectrolytes on top of fixed erythrocyte cells as templates. TEM imaging revealed a surface structure composed of GO sheets when the GO assembly was performed with a high GO concentration. The presence of GO was confirmed by Raman spectro-microscopy that revealed the presence of the GO in the capsule structure. AFM revealed that the capsule walls are thin films with a thickness of approx. 15 nm. Our result shows an alternative method for the fabrication of capsules entailing GO in a dense arrangement that could eventually have applications in drug delivery and optoelectronics by developing hybrid thin polymer films containing graphene oxide or its reduced form rGO.
